# Nanosecond range electric pulse application as a non-viral gene delivery method: proof of concept

**DOI:** 10.1038/s41598-018-33912-y

**Published:** 2018-10-19

**Authors:** Paulius Ruzgys, Vitalij Novickij, Jurij Novickij, Saulius Šatkauskas

**Affiliations:** 1 Biophysical Research Group, Vytautas Magnus University, Vileikos g. 8-212, 44404 Kaunas, Lithuania; 2 Institute of High Magnetic Fields, Vilnius Gediminas Technical University, Vilnius, Lithuania

## Abstract

Current electrotransfection protocols are well-established for decades and, as a rule, employ long micro-millisecond range electric field pulses to facilitate DNA transfer while application of nanosecond range pulses is limited. The purpose of this paper is to show that the transfection using ultrashort pulses is possible by regulating the pulse repetition frequency. We have used 200 ns pulses (10–18 kV/cm) in bursts of ten with varied repetition frequency (1 Hz–1 MHz). The Chinese Hamster Ovary (CHO) cells were used as a cell model. Experiments were performed using green fluorescent protein (GFP) and luciferase (LUC) coding plasmids. Transfection expression levels were evaluated using flow cytometry or luminometer. It was shown that with the increase of frequency from 100 kHz to 1 MHz, the transfection expression levels increased up to 17% with minimal decrease in cell viability. The LUC coding plasmid was transferred more efficiently using high frequency bursts compared to single pulses of equivalent energy. The first proof of concept for frequency-controlled nanosecond electrotransfection was shown, which can find application as a new non-viral gene delivery method.

## Introduction

Electroporation is a common method to facilitate intracellular delivery of membrane-impermeable molecules^1–3^. The array of applications includes cancer treatment^4–7^, drug delivery^8,9^, gene transfer^10–12^, food processing^13^ and biotechnology^14,15^. Such procedure requires specific pulse parameters (amplitude, duration, number of pulses, etc.) to trigger the desired electroporation effect, which varies between different cell types^16–20^. Recently, a new electroporation modality, which employs nanosecond, high intensity (tens to hundreds of kV/cm) electric field pulses became a subject of intensive investigations^21–23^.

Sub-microsecond or nanosecond range pulsed electric field (ns-PEF) allows countering multiple limitations which exist in conventional micro-millisecond range electroporation. Firstly, ns-PEFs offer better control of delivered energy, while maintaining primarily non-thermal treatment^24–26^. Also, nsPEFs extend the flexibility of electroporation, allowing to induce apoptosis^27^ and non-chemical triggering of Ca^2+^ channels^28^. Additionally, the manipulation of other cell functions is possible due to electric field induced displacement currents and the non-thermal interactions with subcellular structures^29,30^. Lastly, ns-PEF allows to minimize electrochemical reactions^31^ during the procedure and a more patient-friendly treatment with minimal muscle contractions^32^ is possible.

One of the electroporation applications is gene delivery both *in vitro* and *in vivo*^10,12^. Well established protocols for efficient electrotransfection have been already established for decades^33–37^. However, all of them require application of long micro-millisecond range pulses or combination of microsecond and nanosecond bursts to facilitate DNA transfer^38^. At the same time, electrophoresis has a crucial role during transfection^39^. However, not all of the aspects of how the electroporation mediates gene electrotransfer and expression in cells and tissues are known^40^. As a result, electrophoresis is a limiting parameter for application of ultrashort pulses. Nevertheless, new pulse delivery techniques emerge and the variation of pulse repetition frequency (PRF) appears to be a powerful tool for control of electroporation efficiency^40–43^. Low frequency protocols offer the possibility to induce the phenomenon of cell sensitization^44^, while higher frequency range allows to counter bioimpedance problems and reduce muscle contractions^45,46^. However, since the amplification of the external electric field in the cell membrane is frequency dependent and decreases in the sub-megahertz range^47,48^ the MHz region is still poorly focused, thus remaining challenging technologically, and therefore mostly a subject of theoretical analysis.

In our previous work, we have shown that it is possible to generate a high frequency pulse burst to achieve a threshold PRF (presumably unique for each cell type and other electroporation conditions), when the discharging (transmembrane potential relaxation) time of the membrane is longer than the delay time between the pulses^49^. As a result, the response of cells (determined by propidium iodide permeabilization assay) to high PRF was several-fold higher if compared to low frequency protocols. Recently, similar result was confirmed by Semenov *et al*., where authors of the study showed that the uptake of YO-PRO-1 is doubled when the nanosecond pulses are closely spaced^50^.

**Figure 1:**
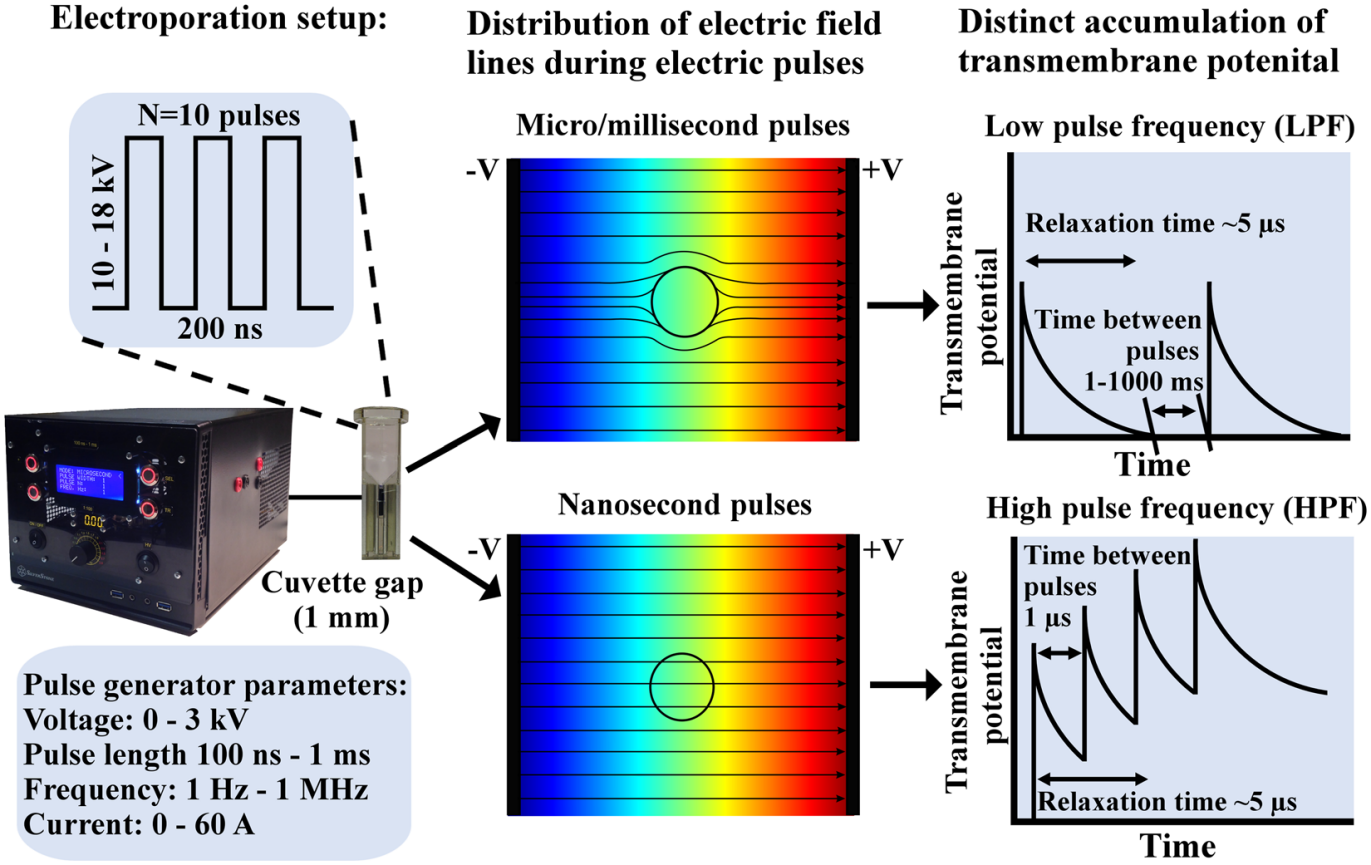
Electroporation setup and the concept for improvement of electrotransfer by means of increase of pulse repetition frequency and resultant transmembrane potential

Taking into the account the membrane charge relaxation phenomenon (in high PRF region) and the capability to induce a more uniform exposure (Fig. 1), we have speculated that it is possible to achieve successful transfection using nsPEF, which has not been shown so far.

In this study, we expand the experimental coverage of high frequency, MHz range nanosecond electroporation, compare the results to conventional low pulse frequency (LPF) protocols and provide a proof of concept for successful frequency-controlled nanosecond electrotransfection.

## Results

### Electrotransfection using GFP coding plasmid

**Figure 2:**
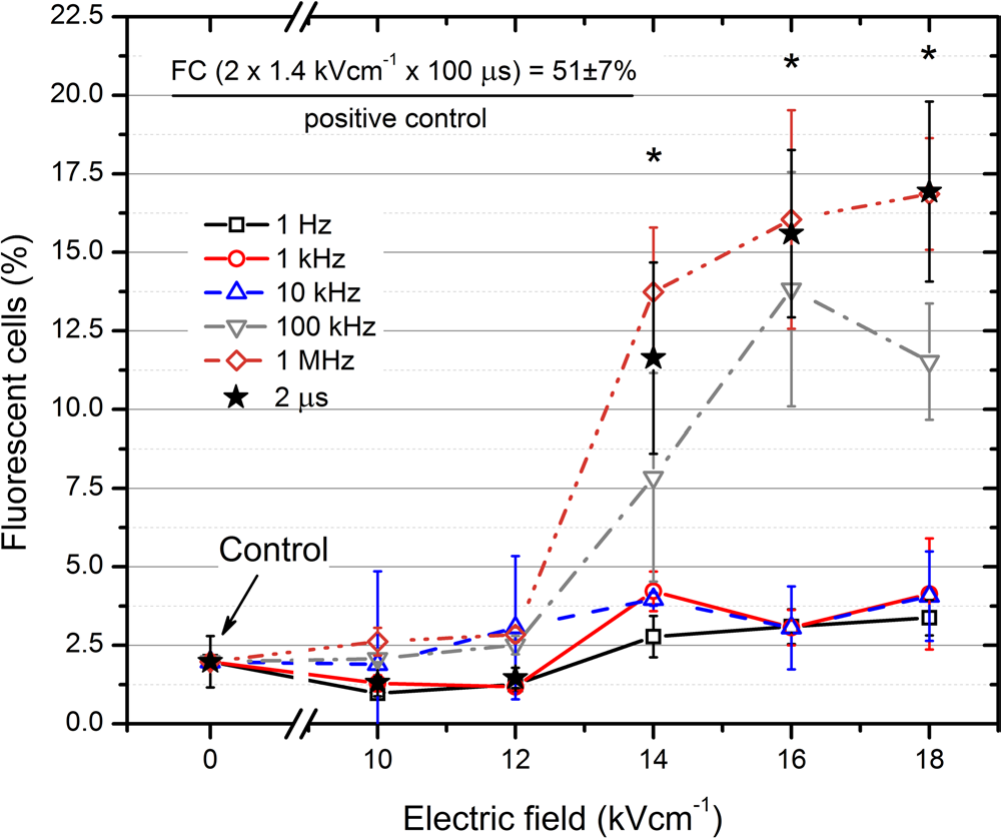
The dependence of the number of fluorescent cells (FC) on the applied PEF treatment parameters. Asterisk (*) represents a statistically significant (P < 0.05) difference compared to the untreated control.

We have evaluated the transfection expression levels of GFP coding plasmid after application of 200 ns × 10 pulses bursts using different PRF protocols and compared the results to a single pulse (2 µs) of identical energy and to the standard 100 µs pulses (2 × 1.4 kV/cm × 100 µs), the latter serving as a positive control. The cells were treated with PEF and the fraction of GFP positive cells was evaluated the next day using flow cytometry. The dependence of the number of fluorescent (GFP positive) cells on the applied pulsing protocol is presented in Fig. 2.

As it can be seen in Fig. 2, the low frequency (<100 kHz) nanosecond pulse protocols were not effective (FC <4%) in the whole range of amplitudes (10–18 kV/cm). However, increase of the PRF to 0.1 and 1 MHz allowed to achieve a transfection efficiency in the 10–20% range, which was comparable with single pulse protocol (2 µs). The 2 × 1.4 kV/cm × 100 µs protocol resulted in up to 51 ± 7% GFP positive cells.

**Figure 3:**
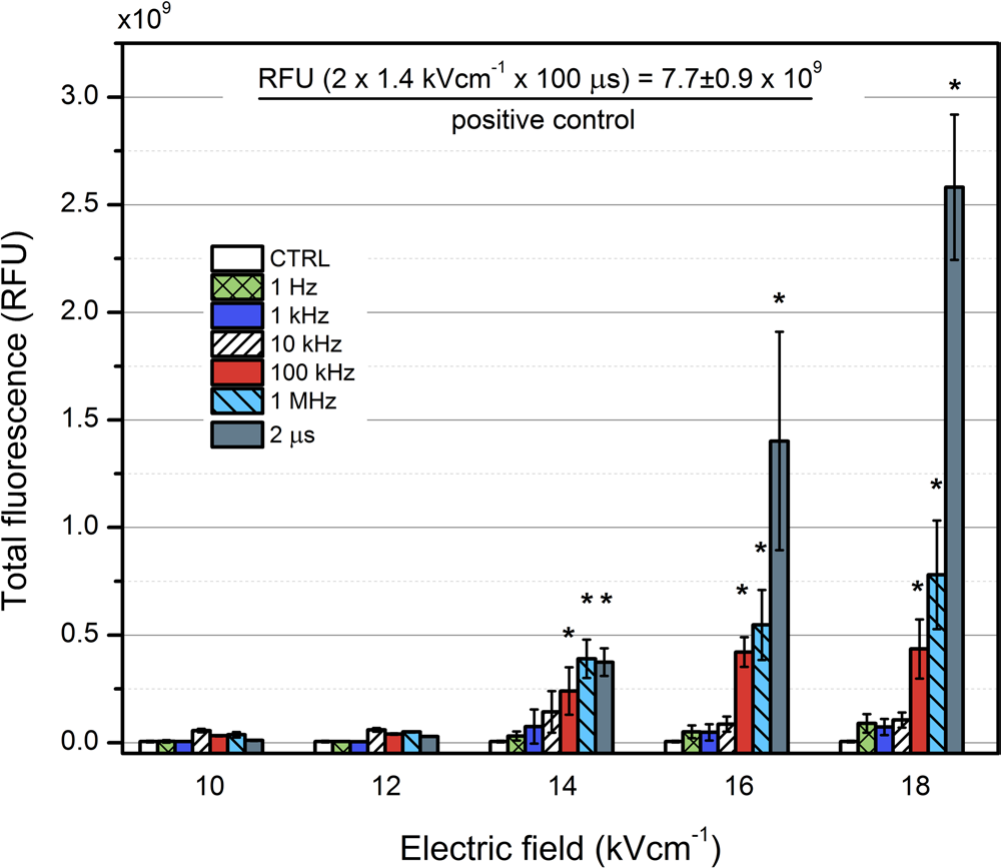
The dependence of total fluorescence on the applied PEF treatment parameters. Asterisk (*) represents a statistically significant (P < 0.05) difference versus untreated control.

Taking into the account that the percentage of GFP positive cells may be manipulated by definition of gates in flow cytometry, we have also evaluated the total fluorescence intensity of the samples in order to quantify the transfection expression levels in terms of relative total GFP production. The results are summarized in Fig. 3.

As it can be seen in Fig. 3 a similar tendency of PRF influence on the electrotransfer persists, however the 2 µs protocol appeared to be significantly superior (2–3 fold) in the 16–18 kV/cm range if compared to the nanosecond pulse protocols in the 16–18 kV/cm range. At the same time, the positive control was more than 10-fold more fluorescent if compared to the highest intensity (18 kV/cm, 1 MHz) nanosecond range protocol.

### Electrotransfection using LUC coding plasmid

**Figure 4:**
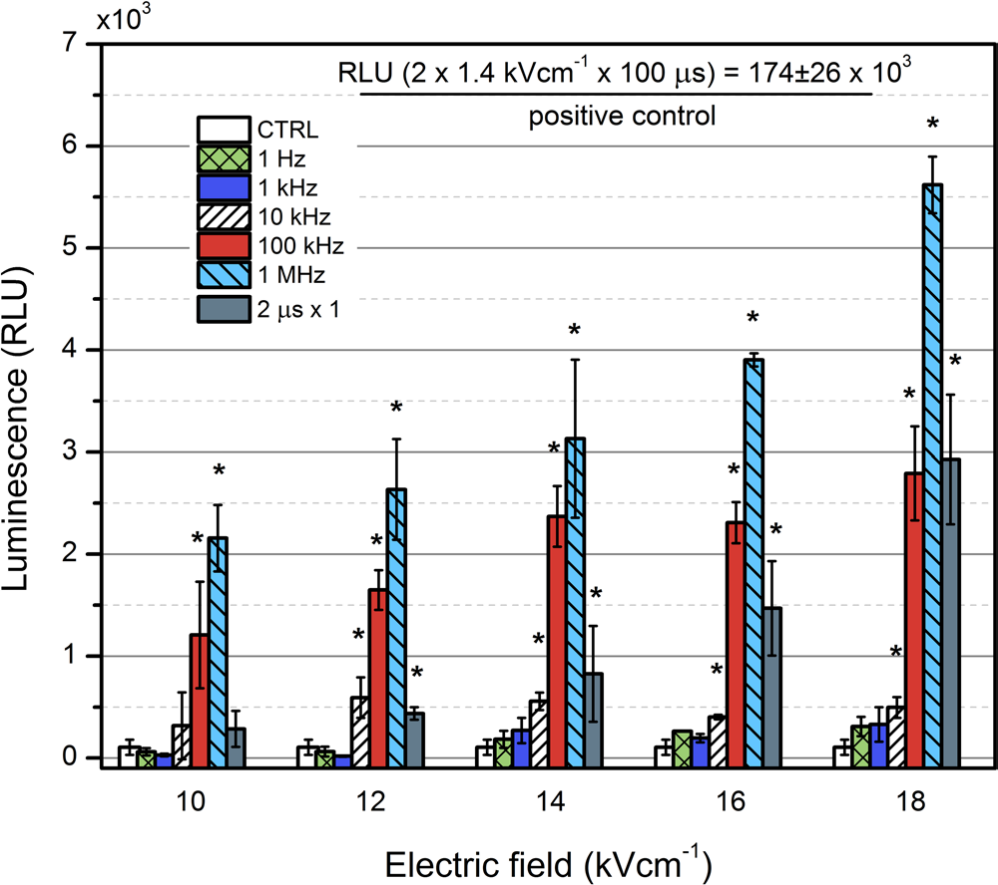
The dependence of the luminescence on the applied PEF treatment parameters. Asterisk (*) represents a statistically significant (P < 0.05) difference versus untreated control.

Similar pulsing and comparison strategy was applied for electrotransfection using LUC coding plasmid. The cells were treated with PEF and the luciferase expression was evaluated after 24 h using luminometer (TECAN GeniosPro). The dependence of sample luminescence on the applied PEF parameters is presented in Fig. 4.

As it can be seen in Fig. 4, the transfection efficiency increased with the increase of PRF. Similar to the case of GFP, the highest expression levels were observed using the 1 MHz protocol, and a dose dependent response was apparent. The 10 kHz protocol was already resulting in low, but statistically significant increase of luminescence. Differently from GFP experiments, the 2 µs protocol was inferior to the high frequency (0.1–1 MHz) bursts in the whole range of investigated amplitudes. However, the positive control had the highest luminescence, which was more than 30-fold higher if compared to the highest intensity (18 kV/cm; 1 MHz) nanosecond pulse burst. Similar to the case of GFP, the low frequency nanosecond range protocols (<10 kHz) did not result in a statistically significant increase of LUC expression levels in the whole range of applied amplitudes.

### Viability

**Figure 5:**
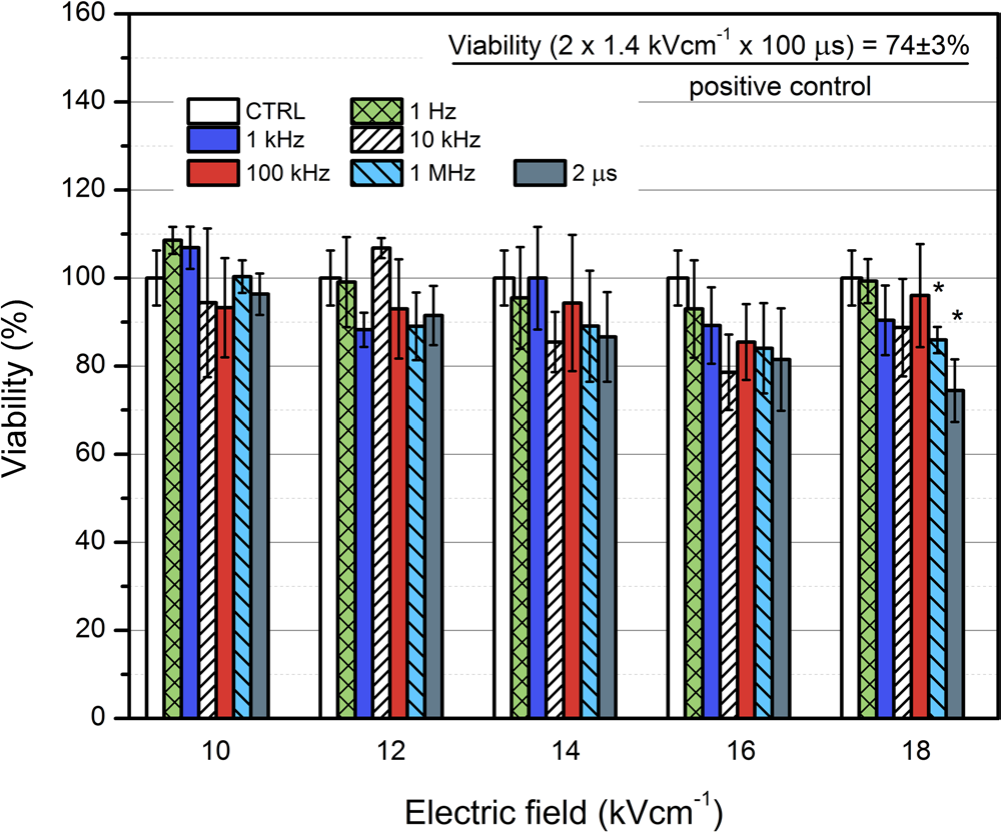
The dependence of cell viability on the applied PEF treatment parameters. Asterisk (*) represents a statistically significant (P < 0.05) difference versus untreated control.

Lastly, the viability of the CHO cells after the PEF treatment using all of the applied protocols was evaluated. The results are summarized in Fig. 5.

As it can be seen in Fig. 5, the viability decrease after the 2 µs pulse was higher compared to the nanosecond pulse protocols, however, it was statistically significant only after the 18 kV/cm treatment. Similar reduction in viability (up to 26%) was also observed in the positive control. All of the applied treatment protocols resulted in a predominantly reversible electroporation of CHO cells.

## Discussion

Electroporation is considered to be an effective non-viral gene delivery approach, which usually requires maintaining of a good balance between electrotransfection efficiency and cell viability^51,52^. The high potential of the gene therapy in clinical applications and the evolution of the whole electroporation area towards shorter pulse duration space^53^ requires development of new and effective gene electrotransfer protocols. Currently, it is believed that nanosecond range PEF are not suitable for transfection^54^, presumably due to lack of sufficient electrophoresis, while the micro-millisecond range pulse protocols are a well-established procedure^33^. Nevertheless, it was shown by Guo *et al*. that pretreating the cells with nsPEF before application of the microsecond range pulses can significantly increase transport of DNA^38^. It implies that the lack of electrophoretic force during nsPEF can be compensated by subsequent microsecond pulses to facilitate DNA electrophoretic transfer. Combined with other nsPEF effects such as cell swelling^55^, organelle membrane modulation^30^ and calcium mobilization^28^, the nsPEF can be effectively used for pretreatment of cells to increase DNA diffusion or stability^38^. However, transfection solely by nanosecond pulse protocols is currently considered unachievable^54^.

In this study, we present a proof of concept that the nanosecond range pulses can be successfully applied for transfection by manipulating pulse repetition frequency.

The high PRF (1 MHz) of nanosecond pulses allowed to achieve significant increase of the transfected cells (up to 17% of cells being GFP positive), which was not possible in 1 Hz–10 kHz range. The phenomenon could be partly justified by the accumulation of charge on the cell membrane when the relaxation time is longer compared to the delay between the pulses, which results in the increase of transmemebrane voltage potential and thus increased permeabilization rate^43,49,50^. However, it does not explain the increased DNA electrotransfer rate, which requires electrophoresis that is limited during short pulses. The hypothesis that the cells see the high frequency burst as a single pulse of equivalent energy^43^ was proposed by Steelman *et al*., however, our data suggests that it is not the only cause of this phenomenon. The LUC coding plasmid was transferred more efficiently using high frequency bursts compared to single pulses of equivalent energy. It could be the cause of the dielectric polarization, which is altered in high frequency bursts since the permittivity parameters are frequency-dependent^56,57^. The effect also could be caused by the increased average pore size and altered polarizability of the cells and/or biomolecules during high frequency bursts. The concept of induction of bigger pore radii during high pulse frequency (HPF) electroporation compared to single pulses of equivalent energy was proposed recently *in silico*^58^. When the PRF increases (or pulse interval decreases), the pore radius also tends to increase^58^, which can explain better DNA electrotransfer. The capability to reorient DNA fragments using high frequency pulsing was highlighted before^59^, while smaller DNA molecules reorient faster than larger DNA molecules, which partially can explain the differences in electrotransfer between LUC and GFP coding plasmids in our study. Lastly, dependent on the inner cell and outer medium conductivities the deformation of the cells during high frequency burst may take place^60^ and requires further study.

Also, in our work we have used the high frequency nanosecond range monopolar pulses with low influence of transient processes, which typically is a technological challenge. Since the electrophoretic transfer of molecules occurs only during the pulse, the prompt reversal of the field (due to transient process or deliberate bipolar pulse) may push them back out of the cell^61,62^. Therefore, in our work the electrophoretic transfer was also preserved during the burst and the cancelation effect was minimized. Application of symmetrical bipolar pulses or high frequency sine wave significantly reduces the efficiency of electroporation in the high PRF range^62,63^. Based on these assumptions, it could be presumed that the high frequency nanosecond range electrotransfection is possible using monopolar bursts only.

It should be noted that our pilot study is a proof of concept and optimization of pulsing parameters (i.e. number of pulses, amplitude, etc.) needs to be performed in the future. The applied MHz range protocols resulted in a predominantly reversible electroporation of CHO cells (>85% of cells survived using the highest intensity burst), therefore, the increase of the treatment energy and PEF amplitude seems possible. Additionally, we were able to observe different trends in transfection expression levels using GFP and LUC coding plasmids. The quantitative analysis revealed that cell transfection using LUC coding plasmid is higher during high frequency bursts compared to single pulses of equivalent energy (Refer to Fig. 4). Notably, it was not the case for GFP (Refer to Fig. 3) and the phenomenon is of particular interest since it may reveal possible differences in the electrotransfection mechanism. The plasmids also vary in size, which can attribute to the electrotransfer rate^63–66^, but the experimental data do not allow to form descriptive conclusions yet, thus, the study of this phenomenon is a matter of future works.

Nevertheless, the acquired data are of high relevance for development of new efficient nanosecond range transfection protocols, while optimization of the protocols and a detailed study of the observed frequency responses is required and will be covered in the future.

## Material and Methods

### Pulsed power setups

The experimental setup consisted of 3 kV, 100 ns^−1^ ms square wave high voltage pulse generator (VGTU, Vilnius, Lithuania)^67^ and a commercially available electroporation cuvette with 1 mm gap between electrodes (Biorad, Hercules, USA). The voltage (V_C_) that was applied to the cuvette was varied in the 0.14–1.8 kV range, corresponding to 1.4–18 kV/cm electric field.

**Figure 6:**
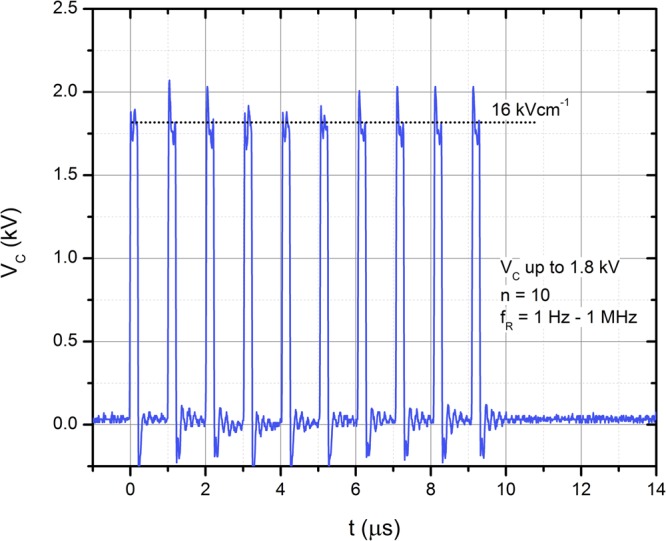
The representative waveform of the high frequency pulse burst. Acquired using DPO4034 digital oscilloscope (Tektronix, Beaverton, USA), post-processed in Origin 8.5 (OriginLab, Northampton, USA).

Three pulsing protocols were used: (1) 10 × 10–18 kV/cm 200 ns pulses of varied PRF (1 Hz–1 MHz); (2) 10–18 kV/cm single 2 µs square-wave pulse of identical energy to account for the effect of PRF and (3) 2 × 1.4 kV/cm × 100 µs (1 Hz) protocol that was used as a positive control for electrotransfection. An example of the high frequency burst waveform of the high frequency burst (10 × 200 ns, 1 MHz) is shown in Fig. 6.

As it can be seen in Fig. 6, the waveform features some overshoots and transient processes, which are due to the specific structure of the pulse forming circuit and the impedance of the load. However, due to very short duration of these oscillations and low voltage of the spikes (up to 300 V at 3 kV), the influence on the transmembrane potential and the results of this study is negligible.

### Biological cells and electroporation

Chinese Hamster Ovary (CHO) cells were used as an electroporation object. Cells where grown in Dublecco’s modified Eagle’s medium (supplemented with FBS (10%), L-Glutamine and penicillin/streptomycin (1%) Sigma, St. Louis, MO, USA) at 37 °C in humidified 5% CO_2_ atmosphere in incubator. After trypsinization, CHO cells where suspended at concentration of 2 × 10^6^ cells/ml in 270 mOsm and 7.1 pH electroporation medium. Electroporation medium was composed of sucrose (242 mM), Na_2_HPO_4_ (5.5 mM), NaH_2_PO_4_ (3 mM) and MgCl_2_ (1.7 mM). The measured specific conductivity of electroporation medium was 0.1 S/m at 25 °C. Cell suspension of 36 µl (2 × 10^6^ cells/ml) in electroporation medium and 4 µl of pmaxGFP (Amaxa, Lonza, Switzerland) or pGL4.13-Luc2/SV40 (Promega, Madison, USA) plasmid at concentration (2 mg/ml) was mixed and put into electroporation cuvette with 1 mm gap between electrodes (Biorad, Hercules, USA). Then cell/plasmid suspension was treated with electric pulses. Afterwards 35 µl of the treaded suspension was taken and transferred into 1.5 ml Eppendorf tube for 10 min incubation at room temperature.

### Flow cytometry and spectrophotometry

Growth medium at volume of 965 μl was added to 35 µl of cell suspension after incubation for 10 min post electroporation. Afterwards 900 μl of diluted cell suspension was transferred into a well of 24 well plate (TPP) and incubated for 24 hours. Then the cells where trypsinized, centrifuged at 200 × g and resuspended in 100 µl of PBS. Then the transfection (GFP positive cells) was evaluated using flow cytometer (BD Accuri C6, BD Biosciences, USA).

For the experiments with luciferase coding plasmid, 950 µl of growth medium was added 10 min post electroporation. Then 139 µl of treated cell suspension (~1 × 10^4^ cells) were plated in the wells of microplate (Plastibrand, Wertheim, Germany) and allowed to grow in cell culture medium for 24 h. Luciferase protein activity was measured with the ONE-GloTM Luciferase Assay System (Promega, Madison, USA) using a luminometer (Tecan GENios Pro; MTX Lab Systems, Vienna, VA).

### Viability assay

Clonogenic assay was performed for cell viability evaluation. 965 μl of growth medium was added to 35 µl of cell suspension after incubation for 10 min post electroporation. Afterwards, 100 μl of diluted cell suspension was taken and mixed with 900 μl of growth medium. Then 55 μl of the suspension was taken and placed in 40 mm diameter Petri dish containing 2 ml of growth medium. This way around 400 cells from each experimental point was grown for colony formation during the period of 6 days. Afterwards, cell colonies were fixed with 70% ethanol for 10 min and stained with crystal violet dye. For evaluation of results cell colonies where scanned with scanner (Canon CanoScan LiDE220). Colonies were counted using open source imaging software ImageJ (National Institutes of Health, USA).

### Statistical analysis

One-way analysis of variance (ANOVA; P < 0.05) was used to compare different treatments. Tukey HSD multiple comparison test for evaluation of the difference was used when ANOVA indicated a statistically significant result (P < 0.05 was considered statistically significant). The data was post-processed in OriginPro software (OriginLab, Northhampton, MA, USA). All experiments have been performed at least in triplicate and the treatment efficiency was expressed as mean ± standard deviation.

## Data Availability

Derived data supporting the findings of this study are available from the corresponding author V.N. on request.
